# Prognostic Value of C-Reactive Protein–Albumin–Lymphocyte (CALLY) Index for Survival in Nivolumab-Treated Metastatic Renal Cell Carcinoma

**DOI:** 10.3390/medicina62061009

**Published:** 2026-05-22

**Authors:** Ali Fuat Gürbüz, Mehmet Zahid Koçak, Oğuzhan Yıldız, Ömer Genç, Bahattin Engin Kaya, Talat Aykut, Melek Karakurt Eryılmaz, Murat Araz, Mehmet Artaç

**Affiliations:** Department of Medical Oncology, Necmettin Erbakan University, Konya 42080, Turkey; mehmetzahidkocak@hotmail.com (M.Z.K.); dr.oguzhan@outlook.com (O.Y.); omergenc58@hotmail.com (Ö.G.); md.enginkaya@gmail.com (B.E.K.); talat_aykut@hotmail.com (T.A.); drangelkarakurt@hotmail.com (M.K.E.); zaratarum@yahoo.com (M.A.); mehmetartac@yahoo.com (M.A.)

**Keywords:** CALLY index, renal cell carcinoma, nivolumab, immunotherapy, prognostic biomarker

## Abstract

*Background and Objectives*: Metastatic renal cell carcinoma (mRCC) remains a lethal disease despite advances with immune checkpoint inhibitors such as nivolumab. However, a substantial proportion of patients exhibit primary resistance or early progression, highlighting the need for reliable and easily accessible prognostic biomarkers. The C-reactive protein–albumin–lymphocyte (CALLY) index is a novel immunonutritional biomarker integrating systemic inflammation, nutritional status, and immune competence. *Materials and Methods*: In this retrospective single-center study, 91 patients with mRCC treated with nivolumab were analyzed. Patients were stratified into low and high CALLY index groups based on a receiver operating characteristic-derived cut-off (0.322). Survival outcomes were assessed using Kaplan–Meier analysis and Cox regression models. *Results*: Patients with a low CALLY index demonstrated significantly shorter progression-free survival (4.5 vs. 13.5 months, *p* < 0.001) and overall survival (9.1 vs. 25.5 months, *p* = 0.003). Multivariate analysis confirmed the CALLY index as an independent prognostic factor for both progression-free survival (HR: 2.63, *p* = 0.002) and overall survival (HR: 1.88, *p* = 0.035). *Conclusions*: The CALLY index is a simple, cost-effective, and reproducible biomarker that independently predicts survival in nivolumab-treated mRCC. It may serve as a practical tool for risk stratification and personalized treatment planning in the immunotherapy era.

## 1. Introduction

Metastatic renal cell carcinoma (mRCC) is a highly lethal malignancy for which therapeutic strategies have been transformed by the advent of immune checkpoint inhibitors (ICIs). Nivolumab, a programmed death-1 (PD-1) inhibitor, improved overall survival compared with everolimus in previously treated mRCC in the CheckMate 025 trial and is now an established standard of care in this setting [1]. Nevertheless, a substantial proportion of patients either fail to respond or experience early disease progression under ICI therapy, underscoring the unmet need for robust, easily accessible biomarkers to stratify prognosis and guide treatment decisions [2,3,4].

Cancer-related inflammation, nutritional status, and host immunity are critical determinants of tumor progression and response to immunotherapy. Peripheral blood-derived indices such as the neutrophil-to-lymphocyte ratio (NLR), platelet-to-lymphocyte ratio (PLR), systemic immune–inflammation index (SII), systemic inflammation response index (SIRI), and pan-immune–inflammation value (PIV) capture aspects of systemic inflammation and have shown prognostic relevance across multiple solid tumors, including RCC treated with ICIs [5,6,7]. However, these scores largely focus on inflammatory cell subsets and only partially reflect the intertwined roles of systemic inflammation, nutritional reserves, and immune competence in shaping ICI outcomes [8].

The C-reactive protein–albumin–lymphocyte (CALLY) index has emerged as a novel immunonutritional biomarker that integrates C-reactive protein (CRP) as a marker of systemic inflammation, serum albumin as an indicator of nutritional and inflammatory status, and lymphocyte count as a surrogate of antitumor immune function, thereby providing a multidimensional assessment of the host–tumor interaction [9]. Evidence specifically linking the CALLY index to ICI outcomes is still limited but growing. In metastatic melanoma, a low pretreatment CALLY index independently predicted worse progression-free and overall survival under anti-PD-1 therapy [10]. In recurrent/metastatic head and neck squamous cell carcinoma and in heterogeneous RCC cohorts, the CALLY index has also outperformed several other systemic inflammation- and nutrition-based scores in nivolumab-treated populations [11,12]. However, its prognostic significance in mRCC patients receiving nivolumab monotherapy has not yet been defined.

We hypothesized that a low pretreatment CALLY index is associated with shorter progression-free survival (PFS) and overall survival (OS) in patients with mRCC treated with nivolumab.

## 2. Materials and Methods

This study is a single-center retrospective cohort study in which patients diagnosed with metastatic renal cell carcinoma and treated with nivolumab were evaluated. A total of 91 patients who were followed at Necmettin Erbakan University between November 2018 and June 2025, had histopathologically confirmed metastatic renal cell carcinoma, received nivolumab treatment, and had complete clinical, laboratory, and follow-up data were included in the study.

Patients were divided into two groups according to pretreatment CALLY index levels designated as low (n = 50) and high (n = 41). Patients with active infection, those who received blood transfusion prior to treatment, or those who were using systemic corticosteroids were excluded from the study.

Demographic characteristics (age, sex), clinical characteristics (ECOG performance status, smoking and alcohol use, body mass index), pathological characteristics (histological subtype, sarcomatoid features, tumor necrosis, lymphovascular invasion, surgical margin, capsule invasion, pathological nodal stage), treatment-related characteristics (presence of primary tumor surgery, presence of metastasis at diagnosis, first-line treatment type and response, nivolumab treatment line, nivolumab response), and prognostic risk scores (MSKCC risk score, IMDC risk score) were retrospectively obtained from the hospital information system and laboratory records.

The CALLY index was calculated using C-reactive protein and albumin levels according to the following formula: CALLY index = Serum albumin level (g/dL) × absolute lymphocyte counts (10^9^/L)/CRP (mg/dL) × 10^4^. CRP values were originally measured in mg/L and converted to mg/dL (1 mg/dL = 10 mg/L) to ensure consistency. For example, a patient with albumin 3.5 g/dL, lymphocyte count 1.5 × 10^9^/L, and CRP 20 mg/L (2 mg/dL) would have a CALLY index of 2.625. The laboratory parameters used in the calculation were obtained from the measurements closest to the time before surgery or nivolumab treatment.

Tumor response and disease progression were assessed according to the Response Evaluation Criteria in Solid Tumors (RECIST) version 1.1. Radiological imaging was performed every 8–12 weeks as part of routine clinical practice, and assessments were conducted by treating physicians. PFS was defined as the time from initiation of nivolumab treatment to documented disease progression or last follow-up, while OS was defined as the time from treatment initiation to death from any cause or last follow-up.

The study was conducted in accordance with the Declaration of Helsinki and relevant ethical guidelines. Due to the retrospective nature of the study, informed consent was not obtained, and ethical approval was granted by the Ethics Committee of Necmettin Erbakan University (approval number: 2025/6142).

### Statistical Analysis

Descriptive statistics were performed using SPSS for Windows version 22.0 (IBM Corp., Armonk, NY, USA). Categorical variables were expressed as number and percentage (%), while continuous variables were presented as mean ± standard deviation or median (IQR). The normality of data distribution was assessed using the Shapiro–Wilk test. Comparisons between CALLY index groups (low vs. high) were performed using Pearson’s chi-square test or Fisher’s exact test when expected frequencies were less than 5 for categorical variables. For continuous variables, the independent samples *t*-test was used for normally distributed variables, and the Mann–Whitney U test was used for non-normally distributed variables. Receiver operating characteristic (ROC) analysis was performed to evaluate the diagnostic performance of the CALLY index in predicting survival. The area under the curve (AUC) was calculated and presented with 95% confidence intervals. The optimal cut-off value was determined using the Youden index (sensitivity + specificity − 1). Based on this cut-off value, patients were divided into low and high CALLY index groups. The Kaplan–Meier method was used to compare PFS and OS between groups. Differences between survival curves were evaluated using the log-rank test. PFS was defined as the time from initiation of nivolumab treatment to disease progression or last follow-up. OS was defined as the time from treatment initiation to death or last follow-up.

Univariate and multivariate Cox proportional hazards regression analyses were performed to determine factors affecting PFS and OS. Variables with *p* < 0.10 in univariate analysis were considered for inclusion in multivariate models to avoid model overfitting given the limited sample size and event number. Multivariate models were adjusted for age group, sex, body mass index (BMI) group, presence of metastasis at diagnosis, primary tumor surgery, MSKCC risk score, IMDC risk score, ECOG performance status, and CALLY index. Results were presented as hazard ratios (HRs) with 95% confidence intervals (CIs). The proportional hazards assumption was tested using log-minus-log plots and Schoenfeld residuals. All statistical tests were two-sided, and a *p*-value < 0.05 was considered statistically significant.

## 3. Results

### 3.1. Baseline Characteristics

In this study, the prognostic value of the pretreatment CALLY index for progression-free survival and overall survival was investigated in patients with metastatic renal cell carcinoma receiving nivolumab. A total of 91 patients were divided into two groups according to CALLY index levels designated as low (n = 50) and high (n = 41). Significant differences were observed between CALLY index groups in terms of several clinical and pathological parameters (Table 1). The proportion of patients with ECOG performance status ≥ 2 was significantly higher in the low CALLY index group compared to the high CALLY index group (42.0% vs. 17.1%, *p* = 0.010). BMI distribution differed significantly between groups (*p* = 0.004), with a higher proportion of patients with BMI < 25 in the low CALLY group (56.0% vs. 22.0%). No statistically significant differences were observed between the groups in terms of other clinical and pathological parameters, including age, sex, smoking and alcohol use, histological subtype, sarcomatoid features, tumor necrosis, lymphovascular invasion, surgical margin, capsule invasion, pathological nodal stage, MSKCC risk score, IMDC risk score, first-line treatment response, first-line treatment type, nivolumab treatment line, and nivolumab response (*p* > 0.05).

Baseline laboratory parameters were compared between the low and high CALLY index groups. Although numerical differences were observed in inflammatory and nutritional laboratory parameters between groups, these differences did not reach statistical significance, possibly due to limited sample size and variability within the cohort (Table 2).

The presence of metastasis at diagnosis was significantly more frequent in the low CALLY index group compared to the high CALLY index group (74.0% vs. 43.9%, *p* = 0.003). In contrast, the proportion of patients who underwent primary tumor surgery was significantly higher in the high CALLY index group compared to the low group (92.7% vs. 50.0%, *p* < 0.001).

### 3.2. ROC Analysis

In ROC analysis, the area under the curve (AUC) for the CALLY index was 0.655 (95% CI: 0.534–0.776), which was statistically significant (*p* = 0.020). The optimal cut-off value determined by the Youden index was 0.322. At this cut-off value, the sensitivity was 70.4% and the specificity was 61.0%. According to this cut-off, patients were categorized into low (n = 50) and high (n = 41) CALLY index groups.

### 3.3. Survival Analysis

**Figure 1:**
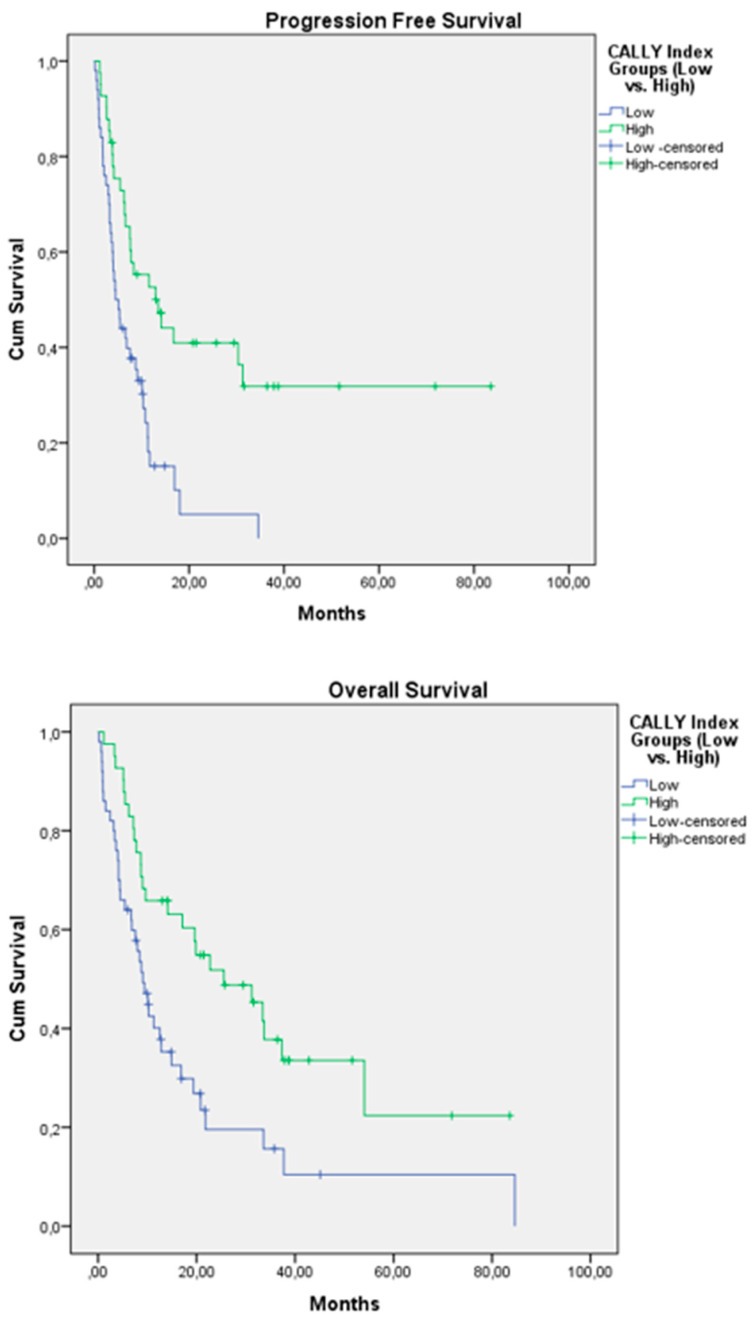
Kaplan–Meier survival curves for progression-free survival (PFS) and overall survival (OS) according to CALLY index groups. Patients were stratified into low and high CALLY index groups based on a receiver operating characteristic (ROC)-derived cut-off value of 0.322. The low CALLY group demonstrated significantly shorter PFS and OS compared with the high CALLY group. Differences between survival curves were assessed using the log-rank test. Median survival times with 95% confidence intervals are presented.

Kaplan–Meier survival analysis demonstrated that the median progression-free survival was 4.50 months (95% CI: 2.83–6.17) in the low CALLY index group and 13.50 months (95% CI: 5.34–21.66) in the high CALLY index group. The difference between groups was statistically significant (log-rank *p* < 0.001), indicating significantly shorter PFS in patients with low a CALLY index. In overall survival analysis, the median OS was 9.10 months (95% CI: 6.33–11.87) in the low CALLY index group and 25.53 months (95% CI: 11.87–39.19) in the high CALLY index group. The difference between groups was statistically significant (log-rank *p* = 0.003), indicating significantly shorter OS in patients with a low CALLY index (Figure 1).

### 3.4. Cox Regression Analysis

Univariate and multivariate Cox regression analyses for PFS are presented in Table 3. In univariate analysis, patients with BMI between 25 and 30 had significantly longer PFS (HR: 0.58; 95% CI: 0.34–0.99; *p* = 0.045), whereas patients with a low CALLY index had significantly shorter PFS (HR: 2.53; 95% CI: 1.51–4.25; *p* < 0.001). In multivariate analysis, after adjustment for BMI group, primary tumor surgery, MSKCC risk score, IMDC risk score, and CALLY index, a low CALLY index remained an independent predictor of PFS (HR: 2.63; 95% CI: 1.44–4.80; *p* = 0.002). Other variables were not independently significant.

Univariate and multivariate Cox regression analyses for OS are presented in Table 4. In univariate analysis, patients with BMI between 25 and 30 had significantly longer OS (HR: 0.56; 95% CI: 0.32–0.98; *p* = 0.043), whereas patients with a low CALLY index had significantly shorter OS (HR: 2.14; 95% CI: 1.28–3.58; *p* = 0.004). In multivariate analysis, after adjustment for age group, BMI group, MSKCC risk score, IMDC risk score and CALLY index, a low CALLY index remained an independent predictor of worse OS (HR: 1.88; 95% CI: 1.05–3.39; *p* = 0.035). Other variables were not independently significant.

## 4. Discussion

In this study, the CALLY index was identified as an independent prognostic biomarker for both progression-free and overall survival in patients with mRCC treated with nivolumab. Patients with a low CALLY index had significantly shorter PFS and OS, and this association remained robust after adjustment for established prognostic factors, indicating that the CALLY index may serve as a clinically meaningful risk-stratification tool in the immunotherapy-treated mRCC population. Although the ROC analysis demonstrated only modest discriminative ability (AUC = 0.655) with relatively low specificity, this finding does not necessarily undermine the clinical relevance of the CALLY index. Prognostic biomarkers in oncology often provide value in risk stratification rather than in binary classification. Therefore, the consistent association between a low CALLY index and inferior survival outcomes suggests that its primary utility lies in identifying high-risk patients rather than serving as a stand-alone predictive tool. Importantly, the direction of this association was consistent across all analyses, confirming that a low CALLY index is associated with worse survival outcomes.

The biological rationale underlying these findings is supported by the central role of systemic inflammation, nutritional status, and host immunity in cancer progression and immunotherapy response. Chronic inflammation promotes tumor growth through cytokine-mediated pathways, enhances angiogenesis, and facilitates immune evasion [13]. Systemic inflammatory signaling, including IL-6-driven pathways, has been implicated in resistance to immune checkpoint blockade [14]. Circulating neutrophils and platelets contribute to tumor proliferation, angiogenesis, and metastatic dissemination, whereas lymphocytes are pivotal for effective antitumor immune surveillance and for mounting de novo responses to ICIs [15]. Consequently, peripheral blood-based inflammatory biomarkers have gained considerable attention as prognostic tools across multiple malignancies, including mRCC treated with nivolumab [16]. Conventional indices such as NLR and PLR provide partial information on systemic inflammation, whereas more complex indices such as SII, SIRI, and PIV attempt to more comprehensively capture immune–inflammatory dynamics [17].

Within this context, the CALLY index offers a distinct conceptual advantage by integrating three biologically complementary domains: systemic inflammation (CRP), nutritional status (albumin), and immune competence (lymphocyte count). The CALLY index has been explicitly characterized as an immunonutritional or immune–nutrition index that reflects inflammation, nutrition, and immunity in cancer patients [18]. Hypoalbuminemia reflects both malnutrition- and inflammation-driven catabolism and has consistently been associated with adverse oncologic outcomes. Lymphopenia indicates impaired cellular immunity, which is particularly relevant in the setting of immune checkpoint inhibition, where peripheral and systemic lymphocyte pools are required for effective antitumor responses. CRP serves as a robust surrogate of IL-6-mediated systemic inflammation and has been repeatedly linked with poor outcomes and diminished ICI efficacy [19]. Thus, the composite nature of the CALLY index allows for a more comprehensive representation of the host–tumor interaction than single-dimension biomarkers or purely inflammation-based scores.

Although the individual components of the CALLY index (CRP, albumin, and lymphocyte count) did not reach statistical significance when analyzed separately, all showed numerically consistent changes in the expected unfavorable direction in the low CALLY group. As a composite biomarker integrating inflammation, nutritional status, and immune competence, the CALLY index may capture a cumulative prognostic signal that is not fully reflected by its individual components alone. The limited sample size and interpatient variability may also have reduced the power to detect significant differences in the individual laboratory parameters.

The prognostic value of the CALLY index has been increasingly recognized in diverse clinical settings. Lower CALLY scores have been associated with poorer survival across multiple malignancies. However, given the absence of direct head-to-head comparisons in our study, the CALLY index should be interpreted as a complementary rather than superior prognostic marker. Recent systematic reviews further confirm that a low CALLY index is consistently associated with inferior OS, DFS, RFS, and CSS across digestive cancers and with higher cancer-related mortality in population-based cohorts [20]. In the immunotherapy setting, a low CALLY index independently predicted worse survival and response in metastatic melanoma patients receiving anti-PD-1 therapy [10]. Our results extend these observations to mRCC and, importantly, to a cohort uniformly treated with nivolumab, addressing a gap in the literature regarding CALLY-based risk stratification in RCC patients on ICI monotherapy.

In mRCC, several inflammatory and immunonutritional indices have been evaluated as prognostic markers in the immunotherapy era. The HALP score, which incorporates hemoglobin, albumin, lymphocyte, and platelet counts, has been shown to correlate with survival in patients treated with nivolumab, with low HALP scores associated with significantly worse outcomes [21]. Similarly, the pan-immune–inflammation value (PIV) has emerged as an independent prognostic factor in mRCC patients receiving nivolumab and has also demonstrated prognostic utility in geriatric mRCC treated with TKIs and in other tumor types undergoing immunotherapy [22]. Additional composite indices such as the C-PLAN index, which integrates CRP, LDH, albumin, performance status, and dNLR, have also shown independent prognostic value in nivolumab-treated mRCC [23]. However, most of these scores are predominantly inflammation-driven and do not explicitly incorporate the nutritional domain, which is increasingly recognized as a determinant of both treatment tolerance and immune competence. By integrating inflammatory, nutritional, and immune parameters, the CALLY index may provide a more holistic prognostic signal, potentially explaining its strong performance in our cohort and in other malignancies.

An additional noteworthy finding is that traditional prognostic models such as the MSKCC and IMDC risk scores did not retain statistical significance in our cohort. This may be explained by the fact that these models were developed in the pre-immunotherapy era, where tumor burden and clinical characteristics predominated. In contrast, the CALLY index incorporates systemic inflammation, nutritional status, and immune competence, which are particularly relevant in patients treated with immune checkpoint inhibitors. Similar limitations of pre-ICI era prognostic models have been reported in real-world mRCC cohorts treated with checkpoint inhibitors, where new immunotherapy-era scores outperformed the Heng/IMDC criteria [24]. Moreover, inflammatory-based scores such as the Meet-URO score (built from NLR, IMDC, and bone metastases) have demonstrated improved risk stratification in nivolumab-treated mRCC, underscoring the added value of integrating systemic inflammation into prognostic frameworks [25]. Our results support the hypothesis that clinicopathological models alone may not fully capture the complex, dynamic host–tumor–immune interactions that drive response to ICIs. Incorporation of robust immunonutritional indices such as the CALLY index into existing or novel prognostic algorithms may enhance prognostic precision and facilitate more individualized therapeutic decision-making.

From a clinical perspective, the CALLY index offers several practical advantages. It is calculated from routinely available, low-cost laboratory parameters and can be readily implemented in daily practice without additional resource burden [26]. Identification of high-risk patients based on a low CALLY index could justify closer clinical and radiologic monitoring, early consideration of treatment modification or intensification, and prioritization for enrollment in clinical trials evaluating novel ICI combinations. Additionally, given the links between nutrition, systemic inflammation, and immune function, the CALLY index may help identify patients who could benefit from structured nutritional and supportive interventions alongside systemic therapy [27,28].

Future prospective, multicenter studies are warranted to validate the prognostic value of the CALLY index in mRCC. Integration of CALLY into composite immunotherapy-specific prognostic models and evaluation using time-dependent ROC analyses may provide a more accurate assessment of prognostic performance in time-to-event outcomes and should be explored in future studies.

This study has several limitations. First, its retrospective and single-center design introduces the potential for selection bias and unmeasured confounding. Second, the relatively small sample size limits subgroup analyses and may reduce statistical power. Additionally, the wide confidence intervals observed for certain covariates, particularly the IMDC risk score in the multivariate Cox regression model, suggest potential instability in some model estimates. This likely reflects the relatively limited sample size, sparse event distribution within specific prognostic subgroups, and possible collinearity among clinically related prognostic variables included in the same multivariable model, such as IMDC risk classification, MSKCC score, ECOG performance status, and the CALLY index. Therefore, these estimates should be interpreted cautiously. Third, baseline imbalances between groups, particularly in ECOG performance status and metastatic burden, raise the possibility of residual confounding despite multivariable adjustment. In addition, the use of a conventional ROC approach in time-to-event data has inherent limitations. Finally, the lack of external validation limits the generalizability of our findings.

## 5. Conclusions

This study demonstrates that the CALLY index is a simple, cost-effective, and reproducible immunonutritional biomarker that independently predicts survival outcomes in mRCC patients treated with nivolumab. Patients with a low CALLY index experience significantly shorter PFS and OS and may benefit from closer monitoring and more individualized treatment strategies. By simultaneously integrating inflammatory, nutritional, and immune parameters, the CALLY index provides a biologically sound and clinically practical tool for risk stratification in the immunotherapy era. Future prospective, multicenter studies should validate these findings, define standardized cut-off values, and explore the integration of the CALLY index—alone or in combination with other immune-inflammatory scores—into prognostic models and clinical decision-making algorithms for mRCC and other ICI-treated malignancies.

## Figures and Tables

**Table 1:** Association Between baseline characteristics and CALLY index groups (low vs. high).

Variable	Category	Low CALLY (n = 50)	High CALLY (n = 41)	*p*-Value
Age group	≤65 years	30 (60.0%)	32 (78.0%)	0.075
	>65 years	20 (40.0%)	9 (22.0%)	
Gender	Female	14 (28.0%)	9 (22.0%)	0.509
	Male	36 (72.0%)	32 (78.0%)	
ECOG PS ≥ 2	No	29 (58.0%)	34 (82.9%)	0.010
	Yes	21 (42.0%)	7 (17.1%)	
Smoking status	Never	27 (54.0%)	21 (51.2%)	0.419
	Current	13 (26.0%)	7 (17.1%)	
	Former	6 (12.0%)	10 (24.4%)	
	Unknown	4 (8.0%)	3 (7.3%)	
BMI group	<25	28 (56.0%)	9 (22.0%)	0.004
	25–30	16 (32.0%)	23 (56.1%)	
	>30	6 (12.0%)	9 (22.0%)	
Metastasis at diagnosis	No	13 (26.0%)	23 (56.1%)	0.003
	Yes	37 (74.0%)	18 (43.9%)	
Primary tumor surgery	No	25 (50.0%)	3 (7.3%)	<0.001
	Yes	25 (50.0%)	38 (92.7%)	
Histological type	Clear cell	33 (66.0%)	31 (75.6%)	0.544
	Papillary	8 (16.0%)	7 (17.1%)	
	Other	9 (18.0%)	3 (7.3%)	
MSKCC risk score	Low	12 (24.0%)	17 (41.5%)	0.111
	Intermediate	25 (50.0%)	19 (46.3%)	
	High	13 (26.0%)	5 (12.2%)	
IMDC risk score	Low	8 (16.0%)	15 (36.6%)	0.076
	Intermediate	28 (56.0%)	21 (51.2%)	
	High	13 (26.0%)	5 (12.2%)	
	Unknown	1 (2.0%)	0 (0.0%)	
Best first-line response	Partial response	18 (36.0%)	16 (39.0%)	0.166
	Stable disease	10 (20.0%)	14 (34.1%)	
	Progression	22 (44.0%)	11 (26.8%)	
First-line treatment	Interferon	13 (26.0%)	11 (26.8%)	0.453
	Sunitinib	11 (22.0%)	13 (31.7%)	
	Pazopanib	15 (30.0%)	12 (29.3%)	
	5-FU-based	2 (4.0%)	2 (4.9%)	
	Nivolumab	2 (4.0%)	0 (0.0%)	
	Cabozantinib	6 (12.0%)	1 (2.4%)	
	Other	1 (2.0%)	2 (4.9%)	
Nivolumab line	1st line	6 (12.0%)	2 (4.9%)	0.187
	2nd line	32 (64.0%)	20 (48.8%)	
	3rd line	9 (18.0%)	13 (31.7%)	
	4th line	3 (6.0%)	5 (12.2%)	
	5th line	0 (0.0%)	1 (2.4%)	
Best response to nivolumab	PR	16 (32.0%)	18 (43.9%)	0.269
	SD	14 (28.0%)	13 (31.7%)	
	PD	20 (40.0%)	10 (24.4%)	

ECOG PS: Eastern Cooperative Oncology Group Performance Status; MSKCC: Memorial Sloan Kettering Cancer Center; IMDC: International Metastatic Renal Cell Carcinoma Database Consortium; HR: Hazard Ratio; CI: Confidence Interval; BMI: Body Mass Index; ROC: Receiver Operating Characteristic; PFS: Progression-Free Survival; OS: Overall Survival; CRP: C-Reactive Protein; CALLY: C-Reactive Protein–Albumin–Lymphocyte Index; PR: Partial Response; SD: Stable Disease; PD: Progressive Disease.

**Table 2:** Baseline laboratory parameters according to CALLY index groups.

Variable	Low CALLY (n = 50)	High CALLY (n = 41)	*p*-Value
C-reactive protein (mg/dL), median (IQR)	2.9 (2.7–3.1)	2.2 (1.9–2.5)	0.35
Albumin (g/dL), median (IQR)	3.4 (3.0–3.8)	4.1 (3.7–4.5)	0.12
Lymphocyte (10^3^/µL), median (IQR)	1.2 (0.8–1.6)	1.8 (1.3–2.4)	0.18
Platelets (10^3^/µL), median (IQR)	280 (210–390)	220 (180–300)	0.63
Neutrophil (10^3^/µL), median (IQR)	5.7 (5.0–6.4)	4.9 (4.7–5.1)	0.25

**Table 3:** Univariate and multivariate Cox regression analysis for progression-free survival.

Variable	Category	Univariate		Multivariate	
		HR (95% CI)	*p*-value	HR (95% CI)	*p*-value
Age group	≤65 vs. >65 years	0.77 (0.46–1.28)	0.318	–	–
Gender	Female vs. Male	0.82 (0.46–1.46)	0.502	–	–
BMI group	<25 (ref)	1.00	–	1.00	–
	25–30	0.58 (0.34–0.99)	**0.045**	0.70 (0.38–1.27)	0.235
	>30	0.74 (0.38–1.46)	0.386	1.05 (0.49–2.26)	0.905
Metastasis at diagnosis	No vs. Yes	0.92 (0.56–1.50)	0.738	–	–
Primary tumor surgery	No vs. Yes	1.51 (0.90–2.53)	0.116	0.88 (0.45–1.72)	0.705
MSKCC risk score	Low (ref)	1.00	–	1.00	–
	Intermediate	1.10 (0.64–1.87)	0.735	1.82 (0.59–5.62)	0.297
	High	0.93 (0.44–1.96)	0.840	0.09 (0.01–1.09)	0.059
IMDC risk score	Low (ref)	1.00	–	1.00	–
	Intermediate	1.01 (0.58–1.78)	0.966	0.60 (0.19–1.86)	0.373
	High	0.93 (0.43–2.02)	0.855	8.26 (0.71–95.80)	0.091
ECOG PS ≥ 2	No vs. Yes	0.28 (0.16–1.47)	0.08	–	–
CALLY index	Low vs. High	2.53 (1.51–4.25)	**<0.001**	2.63 (1.44–4.80)	**0.002**

HR: hazard ratio; CI: confidence interval; BMI: body mass index; MSKCC: Memorial Sloan Kettering Cancer Center; IMDC: International Metastatic Renal Cell Carcinoma Database Consortium; ECOG PS: Eastern Cooperative Oncology Group performance status; CALLY: C-reactive protein-to-albumin ratio-based index. Multivariate model was adjusted for BMI group, primary tumor surgery, MSKCC risk score, IMDC risk score, and CALLY index. Statistically significant *p*-values (<0.05) are shown in bold.

**Table 4:** Univariate and multivariate Cox regression analysis for overall survival.

Variable	Category	Univariate		Multivariate	
		HR (95% CI)	*p*-value	HR (95% CI)	*p*-value
Age group	≤65 vs. >65 years	0.62 (0.37–1.04)	0.06	0.69 (0.39–1.23)	0.20
Gender	Female vs. Male	0.92 (0.51–1.64)	0.76	–	–
BMI group	<25 (ref)	1.00	–	1.00	–
	25–30	0.56 (0.32–0.98)	**0.04**	0.65 (0.36–1.18)	0.15
	>30	0.72 (0.36–1.46)	0.35	0.97 (0.43–2.18)	0.94
ECOG PS ≥ 2	No vs. Yes	0.35 (0.21–1.65)	0.21	–	–
Metastasis at diagnosis	No vs. Yes	1.02 (0.62–1.67)	0.94	–	–
Primary tumor surgery	No vs. Yes	1.41 (0.82–2.42)	0.21	–	–
MSKCC risk score	Low (ref)	1.00	–	1.00	–
	Intermediate	0.96 (0.54–1.68)	0.88	1.73 (0.48–6.22)	0.39
	High	0.94 (0.45–1.96)	0.87	0.12 (0.01–1.33)	0.08
IMDC risk score	Low (ref)	1.00	–	1.00	–
	Intermediate	0.84 (0.47–1.51)	0.55	0.50 (0.14–1.76)	0.28
	High	1.05 (0.50–2.20)	0.89	7.20 (0.68–76.01)	0.10
CALLY index	Low vs. High	2.14 (1.28–3.58)	**0.004**	1.88 (1.05–3.39)	**0.03**

HR: hazard ratio; CI: confidence interval; BMI: body mass index; MSKCC: Memorial Sloan Kettering Cancer Center; IMDC: International Metastatic Renal Cell Carcinoma Database Consortium; CALLY: C-reactive protein-to-albumin ratio-based index. Multivariate model was adjusted for age group, BMI group, MSKCC risk score, IMDC risk score, and CALLY index. Statistically significant *p*-values (<0.05) are shown in bold.

## Data Availability

The data that support the findings of this study are available on request from the corresponding author. The data are not publicly available due to privacy or ethical restrictions.

## Abbreviations

The following abbreviations are used in this manuscript:

RCC	Renal Cell Carcinoma
ICIs	Immune Checkpoint Inhibitors
PD-1	Programmed Death-1
NLR	Neutrophil-to-Lymphocyte Ratio
PLR	Platelet-to-Lymphocyte Ratio
SII	Systemic Immune–Inflammation Index
SIRI	Systemic Inflammation Response Index
PIV	Pan-Immune–Inflammation Value
CALLY	C-Reactive Protein–Albumin–Lymphocyte
CRP	C-Reactive Protein
PFS	Progression Free Survival
OS	Overall Survival
PD	Progressive Disease
SD	Stable Disease
PR	Partial Response
HR	Hazard Ratio
CI	Confidence Interval
BMI	Body Mass Index
HALP	Hemoglobin, Albumin, Lymphocyte, Platelet
MSKCC	Memorial Sloan Kettering Cancer Center
IMDC	International Metastatic Renal Cell Carcinoma Database Consortium
ROC	Receiver Operating Characteristic
ECOG	Eastern Cooperative Oncology Group
